# miRNA-Mediated Immune Regulation in Islet Autoimmunity and Type 1 Diabetes

**DOI:** 10.3389/fendo.2020.606322

**Published:** 2020-11-26

**Authors:** Martin G. Scherm, Carolin Daniel

**Affiliations:** ^1^Institute of Diabetes Research, Group Immune Tolerance in Type 1 Diabetes, Helmholtz Diabetes Center at Helmholtz Zentrum München, Munich, Germany; ^2^Deutsches Zentrum für Diabetesforschung (DZD), Munich-Neuherberg, Germany; ^3^Division of Clinical Pharmacology, Department of Medicine IV, Ludwig-Maximilians-Universität München, Munich, Germany

**Keywords:** immune regulation, islet autoimmunity, type 1 diabetes, miRNA, regulatory T cell, biomarker

## Abstract

The important role of microRNAs as major modulators of various physiological processes, including immune regulation and homeostasis, has been increasingly recognized. Consequently, aberrant miRNA expression contributes to the defective regulation of T cell development, differentiation, and function. This can result in immune activation and impaired tolerance mechanisms, which exert a cardinal function for the onset of islet autoimmunity and the progression to T1D. The specific impact of miRNAs for immune regulation and how miRNAs and their downstream targets are involved in the pathogenesis of islet autoimmunity and T1D has been investigated recently. These studies revealed that increased expression of individual miRNAs is involved in several layers of tolerance impairments, such as inefficient Treg induction and Treg instability. The targeted modulation of miRNAs using specific inhibitors, resulting in improved immune homeostasis, as well as improved methods for the targeting of miRNAs, suggest that miRNAs, especially in T cells, are a promising target for the reestablishment of immune tolerance.

## Introduction

In type 1 diabetes (T1D), the loss of immune tolerance to beta cells in the pancreatic islets of Langerhans leads to an immune cell-mediated destruction of these insulin-producing cells. This progressive loss of beta cell mass is associated with insufficient insulin secretion, resulting in hyperglycemia and the risk of severe acute and chronic complications (1). The autoimmune attack in T1D is mainly driven by the infiltration of the pancreas by autoreactive T cells. These cells are normally repressed by Foxp3^+^ regulatory T cells (Tregs), which are critical mediators of immune tolerance in the periphery. Impaired tolerance and consequently autoimmune activation, which are major drivers of T1D pathogenesis, are, among others, caused by impairments in Treg induction, stability, and function. microRNAs (miRNAs) are small non-coding RNAs, which have been recently shown to fine-tune the expression of important genes in various immune cell types, including Tregs and thereby critically add to immune regulation. The broad regulatory potential of miRNAs in the immune system indicates the potential of specific miRNA targeting to interfere with aberrant immune reactions and autoimmunity. In this review, we discuss the complex role of miRNA regulatory networks contributing to the pathogenesis of autoimmune T1D, with a particular focus on Tregs. Furthermore, we address three studies, which reported a direct relationship between the upregulation of T cell-specific miRNAs during the onset of islet autoimmunity and impairments in Treg induction, function, and stability, as well as the therapeutic potential of these recent findings.

## Type 1 Diabetes

### Immune Tolerance

Diseases with an autoimmune etiology are characterized by impaired immune tolerance, which results in an overshooting immune reaction directed against the body’s own healthy cells and tissues. The steady state of the immune system is a complex and precisely regulated balance between immunity and tolerance, which requires the accurate control of various immune mechanisms and cell types. One hallmark of proper immune function is the discrimination between the organism’s own structures and pathogens, enabling the elimination of potentially harmful invaders without affecting own cells and tissues.

Due to its crucial role for proper immune function, this discrimination between self and non-self and the control of tolerance is mediated by two distinct mechanisms: During their development in the thymus, lymphocytes with a high affinity for self-antigens are negatively selected by deletion (apoptotic cell death) or functional inactivation (anergy). This mechanism is called recessive tolerance, and it was first proposed in the clonal selection theory.

Some autoreactive immune cells can circumvent the recessive tolerance and exit the thymus. To prevent fatal autoimmune attacks of these cells in the periphery, a second control mechanism maintains self-tolerance in the periphery. This peripheral tolerance is termed dominant tolerance and it is carried out by a specific T cell lineage called regulatory T (Treg) cells that can actively suppress other immune cells, including autoreactive T cells.

The dysregulation of these important control processes in the periphery promotes the maintenance and activation of autoreactive lymphocytes, which critically drives the development of autoimmune disorders.

### Islet Autoimmunity and Type 1 Diabetes

To date, more than 80 autoimmune diseases have been described. They can be classified into systemic diseases, such as systemic lupus erythematosus and Sjogren’s syndrome, and organ-specific diseases, such as multiple sclerosis and T1D. T1D shows a rising incidence worldwide and is the most common autoimmune disease in children (2).

T1D is characterized by the infiltration of the pancreas by immune cells and the destruction of the beta cells in the islets of Langerhans. The major drivers of the pathogenesis are impaired immune tolerance mechanisms. The beta cells in the islets of Langerhans are crucial for the maintenance of blood glucose homeostasis, by sensing the level of glucose and releasing insulin as required. Once the beta cell mass is reduced, the scarce supply of insulin prevents the body’s tissues from taking up glucose from the blood stream, which is essential for the maintenance of proper tissue functioning and homeostasis. This loss of blood glucose control has been generally lethal until the establishment of insulin replacement therapy. To date, this remains the only way of controlling the disease, as no curative treatments are available. However, even with lifelong insulin supply, precise glycemic control remains challenging, and secondary complications like kidney failure and heart diseases occur regularly (3).

Multiple factors have been shown to be involved in the pathogenesis of T1D, in particular genetic and environmental factors such as diet and exposure to microbes and certain viruses influence the risk of developing the disease. The genetic predisposition is well studied and is, besides others, indicated by an increased risk of up to 10 times in children with a first-degree relative with T1D (4–7). The most robust predictor of genetic risk to develop the disease is the human leucocyte antigen (HLA) class II, with the genotype HLA-DR4, HLA-DQ8, conveying a risk of around 5% of developing T1D, even without a family history of the diseases (7, 8).

Multiple autoantibodies against islet autoantigens, such as insulin (9), the tyrosin phosphatase IA2 (10), glutamic acid decarboxylase (GAD) (11), and zinc transporter 8 (ZNT8) (12), appear before clinical symptoms of T1D arise. This presymptomatic phase of autoimmunity is termed islet autoimmunity and its duration is highly variable (13, 14). The time of progression from the first appearance of islet autoantibodies to clinically overt T1D can range from only several months to more than two decades. This heterogeneity in progression of islet autoimmunity indicates plasticity in immune activation and multiple layers of immune tolerance impairments. A multitude of recent studies focused on drivers of T1D pathogenesis; however, the precise cellular and molecular mechanisms underlying the loss of immune tolerance and their contribution to the highly heterogeneous progression from islet autoimmunity to symptomatic diabetes remain insufficiently investigated.

### Mediators of Beta Cell Destruction

In T1D, immune cell infiltration into the pancreatic islets, termed insulitis, initiates the destruction of the beta cells (15). Interestingly, both the architecture of the islets and the level of insulitis differ remarkably between the human disease and mouse models of T1D like the non-obese diabetic (NOD) mouse, which exhibits much higher numbers of infiltrating cells (16). During insulitis, several immune cell types infiltrate the islets, including T cells, B cells, macrophages, dendritic cells (DCs), and natural killer (NK) cells, with CD8^+^ T cells being the most abundant cell type (17). Besides the islet infiltration by immune cells, insulitis is characterized by an increased expression of HLA-I molecules in the islet cells (15, 18). This hyperexpression is accompanied by the production of interferon and is thought to contribute to the high abundance of CD8^+^ T cells in the pancreatic infiltrates. Despite this high abundance of CD8^+^ T cells, CD4^+^ T cells are critically involved in the pathogenesis of several autoimmune diseases including T1D, multiple sclerosis (19), rheumatoid arthritis (20), and Crohn’s disease (21). In T1D, several subsets of autoreactive, islet-infiltrating CD4^+^ T cells have been identified, including Th1, Th2 (22), Th17 (23, 24), Th9, Th22 (25), and TFH cells (26, 27).

The contribution of multiple immune cell types, including T cells, with both effector and regulatory characteristics, highlights the complex pathogenesis of T1D. Many of the molecular mechanisms underlying aberrant T cell activation and the multiple layers of impaired immune tolerance remain largely unexplored, not least because a multitude of mechanisms are involved in the regulation of T cells. One of these mechanisms are miRNAs, which have been recently highlighted as important regulators in various biological settings, including the immune system (**Table 1**).

**Table 1:** Selected miRNAs involved in immune regulation.

miRNA	miRNA regulation	miRNA target	Effect	Cell type	Organism	Reference
let7i	Up	IGF1R	Decreased induction	Treg	Human	(28)
miR10a	Down	BCL6, NCOR2	Reduced expression of Treg genes	Tregs	Mouse	(29)
miR15a/16	Up	Foxp3	Decreased Foxp3 abundance	Treg	Human	(30)
miR15b/16	Up	Rictor, mTor	Increased induction	Treg	Mouse	(31)
miR17	Up	IKZF4	Decreased frequencies	Treg	Mouse	(32)
miR17	Up	CREB1, TGFBRII	Decreased induction	naive T cells	Mouse	(33)
miR19b	Up	PTEN	Decreased frequencies	Treg	Mouse	(32)
miR21	Up	unknown	Increased Foxp3 abundance	Treg	Human	(34)
miR21	Down	STAT3	Decreased frequencies	Treg	Human	(35)
miR21-3p	Up	–	Correlation islet autoimmunity, progression to T1D	serum	Human	(36)
miR23b	Up	Trail, Trail-R2, Fas, Faslg	Proliferation	CD8+ T cells	Human	(37)
miR23/miR27/miR24	Up	TGFB signaling	Decreased induction	naive T cells	Mouse	(38)
miR23a-3p	Down	DP5, PUMA	Apoptosis	beta cells	Human	(39)
miR23b-3p	Down	DP5, PUMA	Apoptosis	beta cells	Human	(39)
miR24	Down	Foxp3	Increased Foxp3 abundance	Treg	Human	(40)
miR25	Up	TGFB signaling	Decreased suppression	Treg	Human	(41)
miR25	Up	–	Correlation with glycemic control, residual beta cell function	PBMCs	Human	(42)
miR29	Up	Mc11	Reduced insulin mRNA levels, impaired insulin secretion, and induced beta cell apoptosis	beta cells	Human/mouse	(43)
miR29a-3p	Up	–	Correlation islet autoimmunity, progression to T1D	serum	Human	(36)
miR31	Down	Foxp3	Increased Foxp3 abundance	Treg	Human	(34)
miR34a	Up	insulin, proinsulin	Reduction of insulin and proinsulin	beta cells	Human/mouse	(43, 44)
miR92a-3p	Up	KLF2	Decreased induction	Treg	Human/mouse	(45)
miR95	Up	unknown	Increased Foxp3 abundance	Treg	Human	(40)
miR98	Up	Trail, Fas	Proliferation	CD8+ T cells	Human	(46)
miR99a	Up	mTor	Increased induction	Treg	Mouse	(47)
miR100	Up	SMAD2/3	Decreased induction	Treg	Human	(48)
miR101	Up	Ezh2	Autoimmune activation	naive CD4+ T cells	Human/mouse	(49, 50)
miR125a-5p	Down	CXCL13	Decreased frequencies	Treg	Human	(51)
miR125-5p	Up	CCR2	Impaired migration	Treg	Human	(52)
miR126	Down	p85B	Decreased induction	Treg	Human/mouse	(53)
miR142-3p	Up	Tet2	Decreased induction and stability	Treg	Human/mouse	(54)
miR142-3p	Up	Ccl2, Ccl17, Cxcl10	Immune infiltration, beta cell death	beta cells	Human/mouse	(55)
miR142-5p	Up	Ccl2, Ccl17, Cxcl10	Immune infiltration, beta cell death	beta cells	Human/mouse	(55)
miR146a	Up	insulin, proinsulin	Reduction of insulin and proinsulin	beta cells	Human/mouse	(43, 44)
miR146a	Up	anti-apoptotic genes	Apoptosis	beta cells	Human/mouse	(43, 44)
miR146a	Down	–	Correlation with GAD and IA2 antibody levels	PBMCs	Human	(56)
miR146a	Down	STAT1	Decreased suppression	Treg	Human	(57)
miR146a	Down	STAT1	Decreased suppression	Treg	Mouse	(58)
miR146b	Up	TRAF6	Decreased suppression	Treg	Human	(59)
miR149-5p	Down	DP5, PUMA	Apoptosis	beta cells	Human	(39)
miR150	Up	mTor	Increased induction	Treg	Mouse	(47)
miR155	Up	Ccl2, Ccl17, Cxcl10	Immune infiltration, beta cell death	beta cells	Human/mouse	(55)
miR181a-5p	Up	PI3K signaling	Decreased induction	Treg	Human/mouse	(60)
miR182	Up	Foxo1	Decreased frequencies	Treg	Mouse	(61)
miR200a	Up	unknown	Decreased frequencies	Treg	Human	(62)
miR202-3p	Up	Cd247, Ccr7	Immune infiltration	autoreactive T cells	Mouse	(46)
miR210	Down	Foxp3	Increased Foxp3 abundance	Treg	Human	(40)
miR210	Up	Foxp3	Decreased frequencies	Treg	Human	(63)
miR214	Up	PTEN	Increased frequencies	Treg	Mouse	(64)
miR326	Up	Ets-1	Decreased frequencies	Treg	Human	(65)
miR326	Up	–	Correlation with GAD and IA2 antibody levels	PBMCs	Human	(66)
miR425-5p	Up	–	Correlation islet autoimmunity, progression to T1D	serum	Human	(36)
miR590-5p	Up	Trail, Fas	Proliferation	CD8+ T cells	Human	(37)
miR663	Up	TGFB1	Decreased frequencies	Treg	Human/mouse	(67)

## miRNAs: Regulators of the Immune System

### miRNA Basics

miRNAs are small single-stranded non-coding RNAs, which are involved in almost all physiological processes, by precisely fine-tuning the expression of regulatory genes. They are a member of the family of small non-coding RNAs (sncRNAs), which are 20–30 nucleotides long and function via Argonaute (AGO) proteins. sncRNAs can be subdivided in three distinct regulatory families: miRNA, which are most abundant in human tissues, siRNA (small interfering RNA), and piRNA (PIWI-interacting RNA). In the tissues, miRNAs are directly involved in the regulation of tissue homeostasis and function, which is reflected by their distinct tissue-specific expression (68, 69). Besides their high abundance, several other characteristics of miRNAs highlight their broad regulatory potential. To date, the miRNA database MirBase contains about 2600 validated human miRNA sequences (70), but based on the constant identification of new miRNA sequences, the actual number of human miRNAs is predicted to be significantly higher (71). Furthermore, the miRNA-mediated gene regulation is a complex interplay of many miRNAs, regulating the expression of the same mRNA. Vice versa, the majority of target genes contain a multitude of miRNA binding sites, and some of them are highly conserved between species (68, 72, 73).

miRNAs are commonly 22 nucleotides in length, and their biogenesis involves several processing steps, including transcription, nuclear processing, export from the nucleus, and cytoplasmic processing. First, RNA polymerase II binds to the promoter of a miRNA gene and transcribes a stem-loop-shaped miRNA precursor, which is much longer than the mature miRNA and termed primary miRNA (pri-miRNA) (74). pri-miRNAs can contain up to six miRNA precursors (75), which are flanked by specific sequences facilitating their processing by a complex of Drosha (76) and DGCR8 (77, 78). The resulting pre-miRNA is exported into the cytoplasm where it is further processed by Dicer (79), yielding the final miRNA duplex. For gene silencing, one strand is discarded while the other one remains in contact with Dicer and associates to several additional proteins such as AGO. In line with its mode of action, the resulting complex is termed RNA-induced silencing complex (RISC) (80).

The recognition of the respective target is facilitated by complementary base pairing between the miRNA seed sequence and the corresponding binding site of the mRNA. The seed sequence is only six nucleotides long, comprising nucleotides 2 to 7 of the mRNA. While the short seed sequence facilitates the precise binding of the mRNA target, this is not sufficient to induce miRNA mediated silencing of the mRNA. For this, the complementary binding of additional miRNA nucleotides, usually 8 and 13–16, to the mRNA target is required (68). Some miRNAs form clusters, also called families, which are characterized by almost identical seed sequences and consequently similar target genes (72). The majority of miRNA binding sites is conserved between species, and they can be commonly found in the 3’ untranslated region (UTR) or the coding region of the mRNA; however, their abundance in the 3’ UTR is slightly increased (54, 73, 81). Mechanistically, the regulation of gene expression is mediated by the formation of the RISC, which induces translational repression or mRNA degradation (82).

In line with the broad regulatory potential of miRNAs and the high numbers of miRNA targets, the dysregulation of miRNA expression is involved in multiple human diseases, such as autoimmunity, cancer, and neurological diseases (83, 84).

### miRNAs as Biomarkers for Islet Autoimmunity and T1D

Based on the high numbers of miRNAs and their involvement in virtually all biological processes, including immune regulation, multiple studies investigated their biomarker potential in the context of islet autoimmunity and T1D (42, 56, 66, 85–91). In contrast to the majority of disease symptoms, biomarkers enable the objectively quantifiable characterization of a disease and its progression. This is critical for early and precise diagnosis and treatment and facilitates strategies of personalized precision medicine aiming at the maximum benefit for the patient.

#### Cell-Free Circulating miRNAs

Recent studies have investigated levels of circulating miRNAs in blood or serum from T1D patients in order to evaluate their biomarker potential for the prediction of T1D onset and progression. Circulating miRNAs are particularly suitable as biomarkers in clinical practice because their analysis is feasible in small volumes of blood or serum. Several differentially expressed miRNAs regulate both pancreatic beta cells and immune cells (42, 66, 85). For example, the increased abundance of circulating miR25 correlated with glycemic control and residual beta cell function in patients with newly diagnosed T1D (42). Similarly, levels of the miR23~27~24 cluster were increased during disease progression in children with T1D and correlated with osteoprotegerin abundance. Importantly, combining the levels of osteoprotegerin and the miR23~27~24 cluster in plasma of newly diagnosed T1D patients enabled the prediction of insulin secretion 12 months after diagnosis (86). Another study analyzed plasma miRNAs, immune cell subsets, and specific features of T1D and revealed correlations between miRNAs and T1D onset (let7c-5p, let7d-5p, let7f-5p, let7i-5p, miR146a-5p, miR423-3p, miR423-5p), C-peptide levels in the serum (miR142-5p, miR29c-3p), glycated hemoglobin (miR26a-5p, miR223-3p), and ketoacidosis (miR29c-3p). Furthermore, the analysis pointed towards a link between plasma miRNAs and certain immune cell subsets, which was limited to T1D patients (87). In diabetic NOD mice, miR409-3p was reduced in the plasma as well as in islet infiltrates, and miR409-3p levels were associated with insulitis severity. In human patients with recent onset of T1D, plasma levels of miR409-3p were comparably reduced and correlated inversely with the levels of HbA1c (88). A recent work systematically reviewed and analyzed profiles of circulating miRNAs in T1D patients and suggested a combination of 11 miRNAs (miR21-5p, miR24-3p, miR100-5p, miR146a-5p, miR148a-3p, miR150-5p, miR181a-5p, miR210-5p, miR342-3p, miR375, miR1275), which are involved in several facets of immune regulation and beta cell function, as biomarkers for T1D (89).

#### miRNAs in Blood-Circulating Cells

In peripheral blood mononuclear cells (PBMCs), several miRNAs were found to be correlated with T1D. miR21a and miR93, two miRNAs involved in the regulation of apoptosis and inflammation by targeting NF-κB signaling, were significantly downregulated in patients with recently diagnosed T1D (85), while other miRNAs such as miR20a and miR326 were upregulated (90). The analysis of miRNA signatures in PBMCs also revealed associations with autoantibodies in T1D patients, with increased levels of miR326 correlating with GAD and IA2 antibodies (66) and reduced levels of miR146a correlating with antibodies against GAD (56).

#### miRNAs as Biomarkers During Islet Autoimmunity

So far, most studies analyzed miRNA signatures in individuals with established T1D. However, the relevance of islet autoimmunity for the understanding of T1D pathogenesis as well as for the development of intervention strategies suggests the analysis of miRNA expression in this critical presymptomatic phase. The analysis of miRNA profiles in the presymptomatic phase of T1D was conducted in a recent study that analyzed miRNA signatures in the serum of individuals with a high genetic risk for developing T1D and ongoing islet autoimmunity, as indicated by the presence of multiple islet autoantibodies (91). However, the resulting miRNA expression patterns could not distinguish the islet autoimmunity group from individuals with newly diagnosed T1D or healthy individuals and was unable to predict the progression to clinical T1D. Despite these limitations, a set of differentially expressed miRNAs exhibited significant correlations with glycemic status and antibody titers in individuals with islet autoimmunity. Another study investigated signatures of circulating miRNAs in the serum of autoantibody-positive children vs. their autoantibody-negative siblings. In this dataset, several miRNAs, in particular miR21-3p, miR29a-3p, and miR424-5p, correlated with islet autoimmunity and the progression to T1D (36). Despite these important insights, additional evidence is needed to support the concept that circulating miRNAs are a valuable tool for human T1D risk assessment.

#### Limitations and Next Steps

Whole blood or serum samples of T1D patients are readily available, but the potential of miRNA profiles in such samples to reveal the underlying mechanisms of pathogenesis and progression of organ-specific autoimmune diseases is limited by several aspects. Firstly, profiles of circulating miRNAs most likely do not accurately reflect the environment in the organ, which is the target of the autoimmune attack. Secondly, whole blood and PBMCs represent a highly diverse mixture of various immune cell types. It appears likely that changes in the composition of these immune cell subsets or their miRNA expression have a more profound impact on miRNA profiles than global changes in miRNA expression in the blood or serum. Therefore, the analysis of relevant miRNAs in distinct immune cell subsets, with a particular focus on mediators of autoimmunity, such as effector T cells or Tregs, will provide critical advantages in the analysis of mechanisms underlying T1D pathogenesis. Furthermore, the dissection of miRNA signatures in relevant immune cell subsets, directly in the affected organs, appears to be crucial for the validation of miRNA biomarker signatures with the goal to better understand their contribution to immune activation and the progression to clinical T1D. However, the analysis of miRNA profiles in the respective target organ is often impeded by the limited sample availability, especially during the important pre-symptomatic phase. Furthermore, the affected organs generally contain only very low numbers of the relevant immune cells additionally hindering the broad applicability of these in principle promising approaches.

### miRNAs Involved in Immune-Mediated Beta Cell Destruction

To understand the specific contribution of miRNAs to the onset of islet autoimmunity and the progression to T1D, it is crucial to shift the focus from miRNAs as biomarkers to a more mechanistic dissection of their role for the upstream regulation of autoimmune activation and tolerance impairments.

In addition to the broad regulatory impact of miRNAs in various T cell subsets, modulating immune activation and impaired tolerance, their potential to directly drive the destruction of the pancreatic beta cells has been suggested as an additional layer of regulation. In T1D, the beta cells respond to the inflammatory milieu created by immune cell invasion with the activation of several pathways, which can intensify the immune reaction by inducing beta cell dysfunction, apoptosis, and the secretion of proinflammatory cytokines, attracting more immune cells into the islets (92). Several recent studies suggest that these responses are among others mediated by miRNAs (93).

Cytokines that are typically secreted by pancreas infiltrating immune cells directly modulated miRNA expression in a murine beta cell line (94). Beta cells were exposed to IL-1*β*, TNF-*α*, IFN-*γ*, or a combination of these cytokines for 24 h, and the assessment of miRNA expression showed increased expression of miR21, miR34a, and miR146a. Interestingly, a similar miRNA expression pattern was observed in pancreatic beta cells of NOD mice with considerable immune cell infiltration, while these miRNAs were not upregulated in beta cells of mice that did not show any infiltration in the pancreas (43). This indicates that the expression of these three miRNAs is indeed modulated by cytokines released by infiltrating immune cells. Furthermore, the exposure of cultured human islets to IL-1*β* resulted a comparable increase in expression of miR21, miR34a, and miR146a. The analysis of gene expression revealed that this short-term exposure to cytokines resulted in the miRNA-mediated reduction of insulin and proinsulin mRNA, which is also seen in T1D. In contrast, the sustained exposure to a cytokine-induced inflammatory environment induces apoptosis in human and murine beta cells (44). miR34a and miR146a could be directly linked to cytokine-mediated cell death, while the role of miR21 remains controversial. Inhibition of miR34a and miR146a resulted in higher survival rates in murine beta cell cultures exposed to proinflammatory cytokines, while the inhibition of miR21 had the opposite effect. The dissection of this effect demonstrated that during the exposure to inflammatory cytokines, miR21 triggers a protective response, which is mediated by the downregulation of cell death inducer PDCD4 (95). In addition, the exposure to IL-1*β* and IFN-*γ* resulted in differential regulation of 57 miRNAs in cultured human islets. The reduced expression of three miRNAs—miR23a-3p, miR23b-3p, and miR149-5p—upregulated the pro-apoptotic Bcl-2 family members DP5 and PUMA and consequently promoted apoptosis of beta cells (39).

Another study demonstrated a gradual upregulation of the miR29 family during the course of insulitis in NOD mice (96), and this effect was also observed in cultured murine and human islets when exposed to proinflammatory cytokines (43). This upregulation resulted in reduced insulin mRNA levels, impaired insulin secretion, and induced beta cell apoptosis by targeting Mcl1, which is an anti-apoptotic protein and a confirmed target of miR29.

Another mechanism of miRNA-mediated promotion of beta cell death and its contribution to T1D have been described recently. Both murine and human T cells release exosomes containing miR142-3p, miR142-5p, and miR155-5p, which can be transferred to beta cells and induce their apoptosis. The inhibition of these miRNAs in beta cells prevented apoptosis and protected NOD mice from diabetes development accompanied by higher insulin levels, lower insulitis scores, and reduced inflammation in these mice. Mechanistically, the exosomal miRNAs increase the expression of several chemokine genes, including Ccl2, Ccl7, and Cxcl10 in beta cells, promoting immune infiltration and beta cell death (55).

In sum, these studies indicate that miRNAs are important mediators of cytokine-induced beta cell destruction and dysfunction by modulating several different pathways in response to cytokine exposure and are in line with the concept that they can function as communicators between cells of the immune system and pancreatic beta cells.

### miRNA Regulation in T Cells

#### T Cell Development

Dynamic changes of miRNA expression in hematopoietic precursors indicate their importance for the development and differentiation of various subsets of hematopoietic cells, including T cells. Multiple miRNAs can be linked to T cell differentiation, e.g., miR125b whose upregulation correlates with the specification of progenitor cells into the lymphocyte lineage and in later stages contributes to survival and maintenance of these cells in mice (97). Furthermore, miR181a upregulation was shown to be crucial for the development of both murine T and B cells (98, 99).

The involvement of miRNAs in the development of T cells was initially highlighted in mice by the deletion of Dicer, which resulted in impaired CD8 T cell development in the thymus (100, 101). Furthermore, dynamic miRNA profiles could be linked to distinct stages of murine T cell differentiation, suggesting miRNA regulation of thymic T cell development (102). One example is miR181a, which is highly abundant during the CD4^+^CD8^+^ double-positive stage of T cell differentiation. miR181a regulates TCR signaling by increasing the sensitivity to antigenic stimulation (103), and it targets, among others, Bcl2 and CD69, which are involved in positive selection in the thymus, highlighting the critical regulatory role of this miRNA for T cell development.

#### T Cell Function

The activation and proliferation of T cells in response to antigen exposure is a crucial facet of the immune system, which depends on signaling via the TCR and co-stimulatory molecules such as CD28. These signals lead to an upregulation of miR214, which targets PTEN, a negative regulator of T cell activation, resulting in enhanced T cell proliferation in mice (104). Similarly, IL2 signaling upregulates miR182, which, in turn, downregulates Foxo1 in human and murine activated T cells thereby promoting their clonal expansion (105). In contrast, high levels of miR155 and miR221 collectively downregulate PIK3R1, which inhibits human CD4^+^ T cell proliferation and cytokine production (106).

The transcription factor c-Myc, an important regulator of T cell proliferation and apoptosis, executes its function by modulating the miR17~92 cluster in humans and mice (107). C-Myc binds the miR17~92 locus and induces its expression. Two members of this cluster, miR17-5p and miR20a, regulate the expression E2F1 and thereby facilitate precise control of T cell proliferation. In murine Th1 cells, members of the miR17~92 cluster drive the immune response of these cells by targeting PTEN and CREB1, enhancing proliferation and cytokine production, while inhibiting apoptosis (33).

While the miR17~92 cluster is important for the fine-tuning of T cell activation and proliferation, it can also have deleterious consequences when expressed at very high levels. In mice, the high abundance of this cluster is associated with lymphoproliferative disease and autoimmunity, presumably by inhibiting PTEN and the proapoptotic protein Bim (108). Furthermore, by targeting members of important apoptosis pathways, including Bcl2 and Akt/p53, miRNAs can directly regulate T cell apoptosis in the context of human autoimmune diseases and cancer (109, 110).

#### Peripheral T Cell Subsets

Besides their importance for T cell development in the thymus and T cell function, miRNAs are furthermore involved in the differentiation of distinct T cell subsets.

Naive, effector, and memory CD8^+^ T cells exhibit distinct miRNA signatures, both during and after their differentiation. In murine effector T cells, let7f, miR15b, miR142-3p, miR142-5p, miR150, and miR16 are expressed at low levels when compared to the other subsets, while miR21 is upregulated (99). *In vitro* differentiation experiments showed that miR150, miR155, and miRNAs of the let7 family are involved in memory T cell differentiation in mice, among others by targeting KCNIP1 (111). Similarly, human T cell subsets exhibit distinct miRNA patterns. For example, the naive state of human CD4^+^ T cells is maintained by miR125b, which regulates the expression of genes involved in the differentiation to effector cells, such as IFNG, IL2RB, IL10RA, and PRDM1 (112).

Regarding the differentiation into Th subsets, Dicer deficiency in murine CD4^+^ T cells results in a strong bias toward Th1 differentiation, the inability to develop into the Th2 phenotype, and increased IFN-γ production (100). In contrast, miR155-deficient mice exhibit a CD4^+^ T cell compartment, which is strongly shifted towards the Th2 phenotype, including a high abundance of Th2 cytokines, while the function of Th1 cells is altered (113).

Especially the regulation of Th17 cells in autoimmunity has been extensively studied. To date, more than 30 miRNAs have been identified as contributors to function and plasticity of these cells and the Th17/Treg balance in human and murine autoimmunity (114).

#### Autoreactive T Cells

As described above, autoreactive CD4^+^ and CD8^+^ T cells are major drivers of immune infiltration and beta cell damage.

A recent study investigated alterations in T cell gene expression during the development of T1D in NOD mice and revealed the upregulation of several genes involved in auto-reactivity, inducing the infiltration of the pancreas by autoreactive T cell clones. Furthermore, the analysis of miRNA signatures in these cells demonstrated that these changes in gene expression are mediated by differential regulation of miRNAs. For example, miR202-3p targets the Ccr7 chemokine receptor and Cd247, which have been shown to control autoimmunity in NOD mice (46).

Furthermore, a set of miRNAs (miR23b, miR98, and miR590-5p) drives the proliferation of diabetogenic CD8^+^ T cells in T1D patients by downregulating apoptotic genes such as Trail, Trail-R2, Fas, and Faslg (37). Importantly, the forced expression of this set of miRNAs in T cells induced rapid expansion of diabetogenic T cells, indicating that the observed effect is indeed miRNA-mediated.

In islet autoimmunity and T1D naive CD4^+^ T cells can exhibit dysregulated miRNA signatures, which can alter T cell function and bias them toward autoimmune activation. For example, miR101 is upregulated in naive T cells during islet autoimmunity. This miRNA targets Ezh2, leading to a shift toward the Th1 lineage in humans and mice (49, 50).

## Tregs and Their Regulation by miRNAs

### Characterization of Tregs

Although the destruction of the beta cells is mediated by immune cells, in particular T cells, the contribution of T cells to the development of islet autoimmunity is not limited to these autoreactive processes. One major driver of the activation of autoreactive T cells and their invasion into the pancreas are impaired tolerance mechanisms in the periphery. These impairments are particularly the result of aberrations in Tregs, which can directly suppress various immune cells, including autoreactive T cells, making them the main mediators of peripheral immune tolerance (115).

Tregs express the surface markers CD4 and CD25, which is the interleukin 2 receptor α chain. However, since effector T cells also express these surface proteins, the transcription factor Foxp3 is of particular importance for their identification. The high expression of this lineage defining factor is indispensable for differentiation, maintenance, and function of Tregs. (115, 116). Mutations in the Foxp3 gene lead to fatal autoimmune disorders in both humans (IPEX—immunodysregulation, polyendocrinopathy, enteropathy, X-linked syndrome) and mice (scurfy mice), highlighting the critical role of Foxp3 for Tregs and consequently immune homeostasis (117, 118). Similarly, the depletion of Tregs in newborn mice results in impaired immune regulation and severe autoimmune disease (119, 120).

Since its discovery, the major impact of Foxp3 on Tregs has been studied extensively, revealing a complex network of regulatory interactions termed the Foxp3 interactome (121). Specifically, these studies identified the structure of Foxp3 including its functional domains (122–124) and described multiple target genes (125–127) and interactions partners of Foxp3 (128–132). In addition, several studies demonstrated regulatory regions in the Foxp3 promoter and other noncoding sequences of the Foxp3 gene. These regions control the expression of Foxp3 and are regulated by various mechanisms including epigenetic modifications (133, 134) and transcription factor binding (135, 136).

### Tregs in Islet Autoimmunity and T1D

Importantly, several studies analyzing longitudinal samples of children at different stages of T1D showed that these impairments occur during the phase of islet autoimmunity, before the onset of clinical symptoms, indicating that they are critical triggers of autoimmune activation rather than a consequence. Long-term autoimmunity without progressing to symptomatic T1D is associated with increased frequencies of insulin-specific Tregs (45, 137). Furthermore, *in vitro* Treg induction potential of naive T cells from individuals with islet autoimmunity is reduced both at the insulin-specific and polyclonal level (60). These findings illustrate the crucial role of Tregs for the maintenance of immune homeostasis and the potential of boosting Tregs to interfere with autoimmune progression.

The complex regulatory network in Tregs, which is cardinal for immune tolerance, consists of various components, which could be regulated by miRNAs. In fact, several studies have shown miRNA-mediated regulation of Tregs in different stetting, including autoimmunity.

### miRNA Regulation of Tregs

The major impact of miRNAs on Tregs and consequently immune tolerance was indicated by studying the lineage-specific deletion of Dicer and Drosha. The deficiency of these enzymes involved in miRNA processing leads to reduced numbers of thymic and peripheral Tregs and reduced suppressive function resulting in fatal systemic autoimmunity in mice (101, 138–140). Additional studies identified individual miRNAs, which contribute to these defects, by affecting virtually all aspects of Treg biology, including their development, induction, stability, suppressive function, as well as the expression of critical genes.

In T1D patients, high levels of miR125a-5p were found in Tregs isolated from pancreatic lymph nodes, resulting in decreased expression of CCR2 and consequently impaired Treg migration to the pancreas (52). Furthermore, miR510 was shown to be upregulated in Tregs from individuals with T1D, while the levels of miR342 and miR191 were reduced (141). In mice, miR26a levels were reduced in Tregs during T1D progression and the forced expression of this miRNA promoted Treg expansion and suppressed T1D in NOD mice (142).

In line with the complex regulatory network of Foxp3, there are miRNAs, which are involved in Foxp3-mediated regulatory loops. For example, miR155, which is highly expressed in Tregs, is induced by Foxp3 and suppresses signaling pathways, which would interfere with Treg homeostasis. The ablation of this miRNA in mice leads to impairments in Treg development, including decreased levels of Foxp3, which result in reduced frequencies of thymic and splenic Tregs (143, 144). During Treg development in mice, miR155 downregulates SOCS1 (suppressor of cytokine signaling 1) consequently promoting the activity of STAT5, which is critically involved in IL2 signaling and consequently Treg homeostasis (145).

However, the absence of miR155 does not affect the *in vitro* induction or suppressive function of Tregs, as indicated by the ability of miR155-deficient Tregs to prevent autoimmunity in murine transfer models (143).

Retinoic acid promotes the development of Tregs through several mechanisms, some of which are mediated by miRNAs. For example, miR10a-5p, whose expression is restricted to Tregs, is induced by retinoic acid. In mice, miR10a-5p downregulates several effector T cell genes, such as BCL6 and NCOR2, which promotes the expression of Treg-specific genes (29, 146). Although high levels of miR10a-5p correlate with reduced susceptibility to autoimmune diseases in mice, miR10a-5p deficiency does not lead to autoimmunity, suggesting a compensatory effect of other miRNAs with overlapping targets (29, 146).

The importance of miRNAs for efficient Treg induction *in vitro* was shown by impaired Foxp3 expression in murine Tregs induced from Dicer or Drosha deficient naive T cells (101, 138). The investigation of individual miRNAs revealed both inhibitory and promoting effects on *in vitro* Treg induction, and several miRNAs form complex regulatory networks to regulate human and murine Treg induction (47). For example, miR150 and miR99a cooperatively promote Treg induction by targeting mTOR (47), and likewise, miR15a-16 improves Treg induction only in presence of 15b-16 and vice versa (31). In line with the important role of the PI3K/Akt/mTOR signaling for regulating Treg induction vs. T cell activation, this pathway is regulated by several miRNAs, such as miR126 (53). This miRNA contributes to efficient Treg induction in humans and mice by downregulating p85β, which is a regulatory subunit of PI3K, reducing PI3K/Akt/mTOR pathway activity. Accordingly, the inhibition of miR126 impairs Treg induction by increasing PI3K/Akt/mTOR signaling (53). As described above, miR155 targets SOCS1 to promote thymic Treg development. The same mechanisms, promoting STAT5 activity, also support efficient murine Treg induction *in vitro* (147). Besides these miRNAs promoting Treg induction, there are also miRNAs with a negative effect on Treg induction *in vitro* (47). In mice, high levels of two miRNAs of the miR17~92 cluster, miR17 and miR19, interfere with efficient Treg induction while thymic Treg development is unaffected (33). The effect of miR17 is mediated by the targeting of two proteins, which are important for efficient Treg induction: the cAMP-responsive element binding protein 1 (CREB1) and the TGFβ-receptor II. The latter pathway is additionally regulated by the miR23-miR27-miR24 family, which downregulates important components of TGF signaling in mice and consequently impairs Treg induction (38).

## In-Depth Dissection of miRNA Mediated Tolerance Impairments

**Figure 1:**
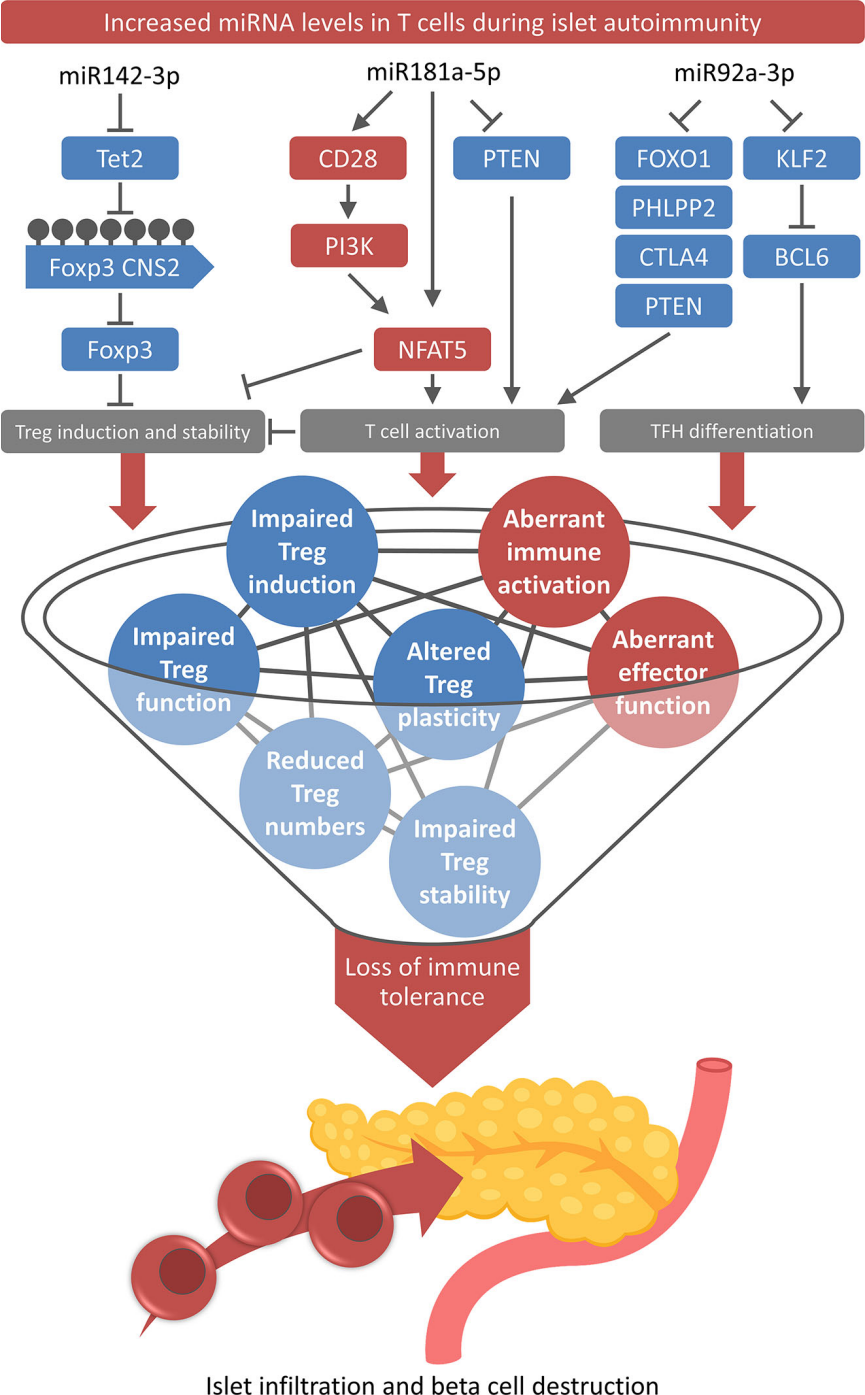
Role of T cell-specific miRNAs for the loss of immune tolerance. High levels of miR-142-3p, miR-181a-5p, and miR-92a-3p and their downstream pathways contribute to multiple layers of tolerance impairments and aberrant immune activation during onset and progression of islet autoimmunity.

As indicated above, several recent studies have investigated the role of specific miRNA in various immune cell subsets, which has significantly driven our understanding of the importance of miRNA-mediated regulation of immune cell differentiation and function as well as immune homeostasis. However, aiming at the ultimate goal of developing future intervention strategies to interfere with aberrant immune activation and delay or even prevent T1D autoimmunity, it is of major importance to understand the underlying mechanisms in more detail. Therefore, the dissection of pathways interfering with immune tolerance and triggering the onset of islet autoimmunity is an important next step following the identification of miRNAs differentially expressed in islet autoimmunity. On this account and given the importance of Treg impairments for insufficient tolerance induction and autoimmune activation, our laboratory has recently investigated the association of individual miRNAs in T cells during the onset of islet autoimmunity and Treg impairments. In three studies, we reported a direct link between dysregulated miRNA expression and defects in Tregs (**Figure 1**).

### miR92a-3p

The first study investigated the role of miR92a-3p in humans and mice, revealing that the T cell-specific increased expression of this miRNA during islet autoimmunity favors the development of T follicular helper (TFH) cell precursors and simultaneously impairs the efficient Treg induction, two mechanisms that can critically contribute to the onset and progression of islet autoimmunity (**Figure 1**) (45).

TFH cells are a subset of CD4^+^ T cells, and their ability to provide support to B cells for the production of high-affinity antibodies makes them an essential part of the humoral immune response (148). TFH cell precursors circulate in the blood where they mediate the induction of antibody responses, suggesting that these cells are an important effector T cell subset involved in the development of autoimmune diseases, which can be mediated by autoantibodies (149, 150). This provided the rationale for the analysis of the role of these cells for the development and progression of islet autoimmunity, which showed increased levels of insulin-specific and polyclonal TFH precursor cells during islet autoimmunity (45). The analysis of differential miRNA expression identified miR92a-3p to be upregulated in T cells from children with recent onset of islet autoimmunity compared to T cells from children with long-term autoimmunity or healthy controls. Furthermore, miR92a-3p expression in T cells directly correlated with the TFH precursor abundance in the peripheral blood. During TFH induction *in vitro*, the specific inhibition of miR92a-3p resulted in decreased TFH cell induction, while a miR92a-3p mimic had the opposite effect. The analysis of known targets of miR92a-3p revealed that high levels of the miRNA resulted in the reduced expression of several negative regulators of T cell activation, including PTEN, PHLPP2, FOXO1, and CTLA4.

Besides the effect on TFH cells, the modulation of miR92a-3p also limits efficient Treg induction by downregulating PTEN and consequently activating the PI3K pathway. These findings indicate that miR92a-3p functions as a shared signaling mediator by controlling negative regulators of T cell activation such as PTEN, which is involved in the control of TFH cells as well as Tregs and their induction. Consequently, *in vivo* high levels of miR92a-3p during the onset of islet autoimmunity were accompanied by reduced frequencies of insulin-specific Tregs and *in vitro* a miR92a-3p mimic impaired efficient Treg induction. The effect of the mimic was reduced upon PI3K inhibition and increased upon PTEN blockade, indicating that these two pathways control the regulation of TFH cell induction vs. Treg induction. Furthermore, KLF2 was identified as a previously unknown target of miR92a-3p. KLF2 promotes S1pr1 expression and BLIMP1 upregulation, which consequently inhibits the TFH master regulator BCL6 (151), thus offering a second mode of action of miR92a-3p to regulate TFH differentiation.

### miR181a-5p

In a second study, we showed that high levels of a specific miRNA in T cells from individuals with recent onset of islet autoimmunity diminish Treg induction capacity in naive CD4^+^ T cells (**Figure 1**). The defective Treg induction occurred in individuals with recent onset but not with long-term autoimmunity without progression to clinical T1D or in the absence of islet autoimmunity.

The impaired Treg induction was accompanied by excessive T cell activation as indicated by higher frequencies and increased proliferation of CD4^+^Foxp3^int^ T cells, which in turn interfered with Treg induction (60).

This enhanced T cell activation could be linked to increased levels of miR181a-5p, which has been identified as a modulator of T cell signaling by fine-tuning the thresholds of antigenic stimulation (152). miRNA modulation *in vitro* demonstrated that high levels of miR181a-5p, induced by a miR181a-5p mimic, resembled Treg impairments during the onset of human and murine islet autoimmunity. Conversely, miR181a-5p inhibition could correct these defects and resulted in higher Treg induction efficacy. The increased expression of miR181a-5p in T cells was accompanied by reduced expression of its direct target PTEN. Low levels of PTEN contribute to excessive T cell activation by enhancing PI3K signaling and increasing abundance and activity of nuclear factor of activated T cells 5 (NFAT5) (153). In addition, high expression of miR181a-5p was accompanied by increased levels of CD28, which is a costimulatory molecule involved in PI3K activation (154), NFAT5 upregulation, and T cell activation. These findings show that miR181a-5p is involved in the regulation of T cell activation vs. Treg induction during islet autoimmunity. High levels of the miRNA downregulate PTEN, which results in higher NFAT5 and CD28 expression, consequently favoring T cell activation and interfering with Treg induction.

In line with these findings, miR181a-5p levels were increased in NOD mice with islet autoimmunity, accompanied by elevated NFAT5 and low PTEN levels, resulting in reduced Treg induction capacity of naive CD4^+^ T cells. The *in vivo* inhibition of miR181a-5p was able to improve islet autoimmunity in these mice, as indicated by reduced levels of pancreas-infiltrating immune cells. This improvement was mediated by increased PTEN levels and decreased expression of NFAT5 and CD28. The important role of NFAT5 was confirmed using NFAT5 deficient mice as well as a NFAT5 inhibitor. Both approaches resulted in improved Treg induction efficiency by upregulating levels of PTEN and of Foxo1, another positive regulator of Treg induction (155).

Given the broad regulatory potential of individual miRNAs, it is most likely that the effect of the miRNAs described above is not limited to islet autoimmunity. In line with this hypothesis, several studies revealed a contribution of miR92a-3p and miR181a-5p to other autoimmune disorders such as neuroinflammation, Th17-mediated inflammation, and lymphoproliferation (32, 108, 156).

### miR142-3p

The third study demonstrated that miRNA-mediated dysregulation of DNA methylation drives islet autoimmunity by interfering with Treg homeostasis in humans and mice (**Figure 1**) (54). Using unbiased approaches including miRNA sequencing and high-throughput sequencing of RNA isolated by crosslinking immunoprecipitation (HITS-CLIP), we identified a set of miRNAs that could contribute to the onset of islet autoimmunity. In this study, we investigated the role of miR142-3p as well as its direct targets and downstream pathways. The reported increased expression of miR142-3p during the onset of islet autoimmunity and its high abundance in the RISC complex, suggesting a critical role of this miRNA in the regulation of CD4^+^ T cells. During early islet autoimmunity, high levels of miR142-3p reduced the expression of an important mediator of DNA demethylation: the methylcytosine dioxygenase Tet2, which was confirmed as a direct target of miR142-3p using a combination of molecular and cellular approaches.

Tet2 belongs to the family of ten-eleven translocation (Tet) methylcytosine dioxygenases that are involved in the regulation of various cellular processes, including the differentiation of CD4^+^ T cells in humans and mice. Tet2 catalyzes the conversion of methylcytosine in the DNA to 5-hydroxymethylcytosine, which is the first intermediate step of DNA demethylation. The demethylation of regulatory regions of the genome facilitates increased binding of transcription factors and consequently the regulation of gene expression. In Tregs, the stable expression of Foxp3 is maintained by the demethylated state of the conserved non-coding sequence 2 (CNS2) within the Foxp3 gene (157–159). As a direct mediator of DNA demethylation, Tet2 is of critical importance for sustained Foxp3 expression and consequently the Treg phenotype. The miR142-3p mediated silencing of Tet2 resulted in higher levels of DNA methylation at the Foxp3 CNS2, which was accompanied by decreased abundance of pancreatic Tregs in islet autoantibody positive NOD mice.

Importantly, the inhibition of miR142-3p *in vitro* and *in vivo* was able to correct the defects resulting from elevated levels of miR142-3p during islet autoimmunity. *In vitro*, miR142-3p inhibition restored Tet2 levels, resulting in improved Treg induction and stability. The application of the miR142-3p inhibitor to islet autoantibody positive NOD mice resulted in increased levels of Tet2, proper DNA demethylation of the Foxp3 CNS2 locus, higher Treg frequencies in the pancreas, and reduced islet autoimmunity. Furthermore, similar patterns were observed in preliminary experiments using humanized mouse models, indicating the relevance of these findings for established human T1D.

In addition to Tet2, the analysis of potential miR142-3p targets revealed several genes that are involved in Treg homeostasis, such as Smad3, TGFβ receptors, and Stat5. TGFβ plays an important role for immune regulation and Treg homeostasis: by phosphorylating Smad proteins, it ensures the expression of Foxp3 (160). Stat5 is induced by IL-2 signaling and is an important regulator of Treg development (161). Like Tet2 signaling, both pathways are involved in the maintenance of Treg homeostasis, by regulating Foxp3 induction and stability, indicating that miR142-3p regulates a complex network of important regulators of Treg differentiation and function.

The results of this study highlight that miR142-3p levels are increased during islet autoimmunity, which, via Tet2 downregulation and aberrant CNS2 demethylation, interferes with Treg induction and stability and consequently drives autoimmune activation. Therefore, the modulation of the miR142-3p/Tet2 signaling pathway, by targeted miR142-3p inhibition or enhancing Tet2 abundance or activity, could be a promising strategy in order to improve Treg induction and stability to interfere with the onset of islet autoimmunity.

## Discussion

**Figure 2:**
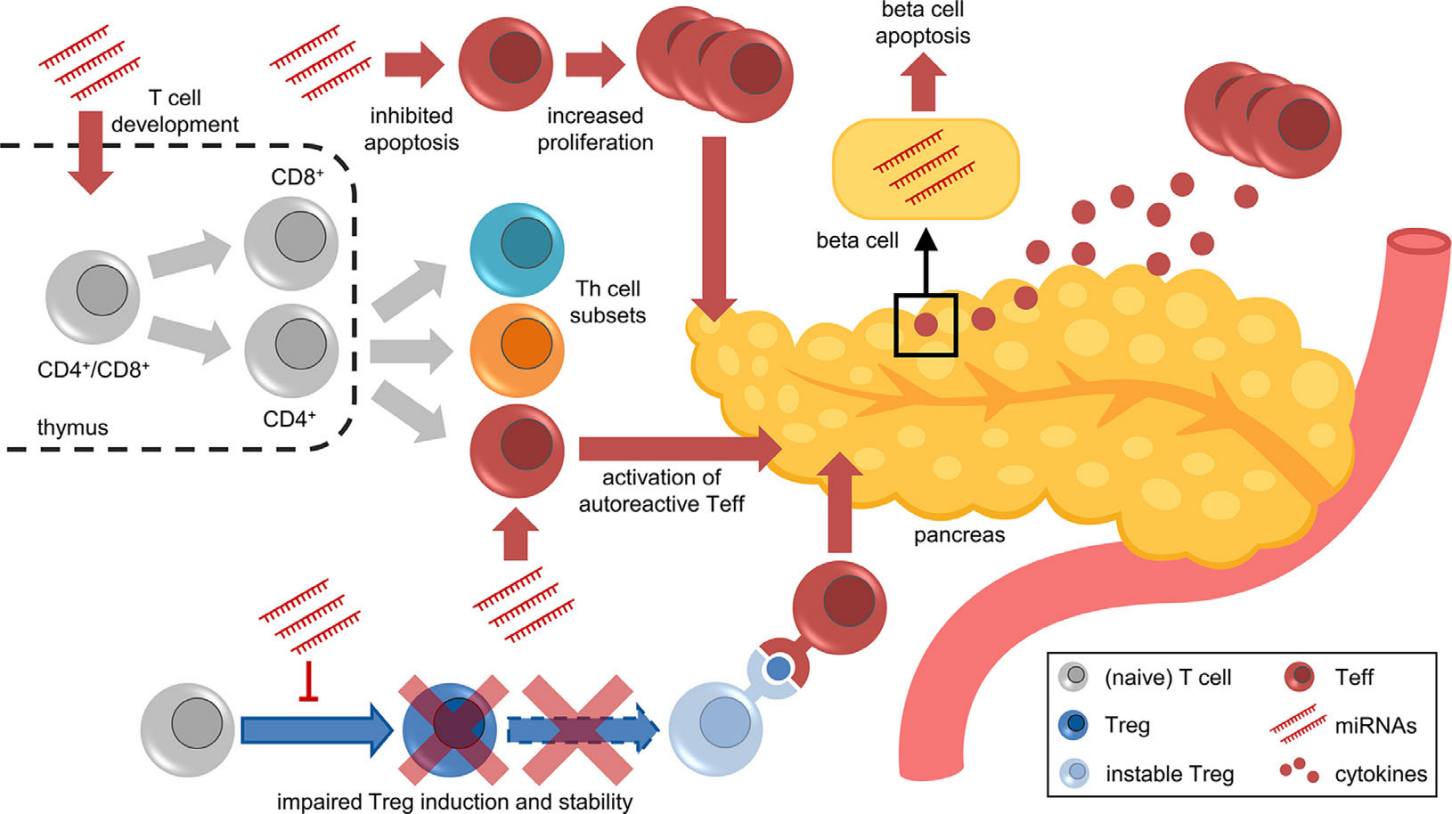
Facets of miRNA-mediated immune regulation in islet autoimmunity and T1D. Aberrant miRNA expression contributes to various aspects of impaired immune regulation, including T cell development in the thymus, differentiation of Th subsets, apoptosis/proliferation of effector T cells, activation of autoreactive T cells, impaired Treg induction and stability, and cytokine-induced beta cell dysfunction and apoptosis.

The highly complex pathogenesis of T1D, which is driven by several immune cell types, including T cells, with both effector and regulatory characteristics, hinders the development of efficient prevention and treatment strategies. Most of the molecular mechanisms underlying aberrant T cell activation and the multiple layers of impaired immune tolerance remain largely unexplored, not least because a multitude of mechanisms are involved in the regulation of T cells. A better understanding of the molecular and cellular mechanisms triggering autoimmune activation and promoting the progression to clinical T1D requires deeper insights into the molecular regulation of important mediators of autoimmunity, such as specific T cell subsets. One of these regulatory mechanisms are miRNAs, which are critically involved in the regulation of proper functioning of the immune system, including T cell development, differentiation, and in particular Treg induction and function (**Figure 2**). For this reason, recent studies investigated T cell-specific miRNAs during ongoing islet autoimmunity and how these miRNAs modulate T cell function and consequently contribute to both activation and progression of autoimmunity. These studies demonstrated that the targeting of individual miRNAs can induce relevant changes in expression of their target genes and modulate downstream signaling pathways, resulting in reduced islet autoimmunity in mouse models.

## Author Contributions

MGS reviewed the literature wrote the manuscript, and prepared the figures. CD reviewed the literature and contributed to the conceptualization of the manuscript. All authors contributed to the article and approved the submitted version.

## Funding

CD holds a professorship grant from the Excellence Program for Outstanding Female Scientists from the Helmholtz Association, is supported by a Research Group at Helmholtz Zentrum München, the German Center for Diabetes Research (DZD),, and through a membership in the CRC1054 of the Deutsche Forschungsgemeinschaft (B11).

## Conflict of Interest

The authors declare that the research was conducted in the absence of any commercial or financial relationships that could be construed as a potential conflict of interest.
